# Manganese Deficiency Is Required for High Itaconic Acid Production From D-Xylose in *Aspergillus terreus*

**DOI:** 10.3389/fmicb.2019.01589

**Published:** 2019-07-09

**Authors:** István S. Kolláth, Ákos P. Molnár, Áron Soós, Erzsébet Fekete, Erzsébet Sándor, Béla Kovács, Christian P. Kubicek, Levente Karaffa

**Affiliations:** ^1^Department of Biochemical Engineering, Faculty of Science and Technology, University of Debrecen, Debrecen, Hungary; ^2^Institute of Food Science, Faculty of Agricultural and Food Science and Environmental Management, University of Debrecen, Debrecen, Hungary; ^3^Institute of Chemical, Environmental and Bioscience Engineering, TU Wien, Vienna, Austria

**Keywords:** *Aspergillus terreus*, D-xylose, itaconic acid, fermentation, specific yield, volumetric yield

## Abstract

Itaconic acid is used as a bio-based, renewable building block in the polymer industry. It is produced by submerged fermentations of the filamentous fungus *Aspergillus terreus* from molasses or starch, but research over the efficient utilization of non-food, lignocellulosic plant biomass is soaring. The objective of this study was to test whether the application of two key cultivation parameters for obtaining itaconic acid from D-glucose in high yields – Mn^2+^ ion deficiency and high concentration of the carbon source – would also occur on D-xylose, the principal monomer of lignocellulose. To this end, a carbon and energy balance for itaconic acid formation was established, which is 0.83 moles/mole D-xylose. The effect of Mn^2+^ ions on itaconic acid formation was indeed similar to that on D-glucose and maximal yields were obtained below 3 μg L^–1^ Mn^2+^ ions, which were, however, only 0.63 moles of itaconic acid per mole D-xylose. In contrast to the case on D-glucose, increasing D-xylose concentration over 50 g L^–1^ did not change the above yield. By-products such as xylitol and α-ketoglutarate were found, but in total they remained below 2% of the concentration of D-xylose. Mass balance of the fermentation with 110 g L^–1^ D-xylose revealed that >95% of the carbon from D-xylose was accounted as biomass, itaconic acid, and the carbon dioxide released in the last step of itaconic acid biosynthesis. Our data show that the efficiency of biomass formation is the critical parameter for itaconic acid yield from D-xylose under otherwise optimal conditions.

## Introduction

Itaconic acid (2-methylene-succinic acid, 2-methylidenebutanedioic acid; IA) is a well-known example of fungal overflow metabolism, the phenomenon of metabolic deregulation caused by a combination of carbon source abundance and serious shortage of phosphate and some micronutrient(s), of which Mn^2+^ ion limitation is the most important one (Batti and Schweiger, 1963; Rychtera and Wase, 1981; Kuenz et al., 2012; Karaffa et al., 2015). The estimated 41.000 t of IA manufactured annually (Geiser et al., 2016) is produced by large-scale submerged fermentations employing the filamentous ascomycete fungus *Aspergillus terreus* (Bafana and Pandey, 2018). Due to the double bond of its methylene group, IA is mainly used as a bio-based, renewable building block in the polymer industry for the production of synthetic fibers, resins, plastics, coatings, and thickeners (Okabe et al., 2009). In principle, it could also replace petroleum-based methacrylic and acrylic acid, thereby opening up a huge market (El-Imam and Du, 2014). However, that would require a significant (up to 25%) fall in prices that currently stands at around $2/kg.

On technical scale, IA (and also citric acid; CA) is mainly produced from molasses or corn starch hydrolysates. However, stiff competition with food applications keeps the fermentation industry searching for cheap, renewable raw materials to be utilized as carbon sources. Hence, research interests over the more efficient utilization of non-food, lignocellulosic plant biomass are soaring for a long time now (Cunha da Cruz et al., 2018). Lignocellulose is a complex polymer of hexose and pentose monomers (Yang et al., 2019), whereby D-xylose is the most abundant pentose (Saha, 2003). However, D-glucose and D-xylose as well as D-xylose and L-arabinose partially interfere with each other’s uptake and metabolism, and thus investigation of their combined conversion will only yield scientifically valid data if the metabolism of these sugars in the absence of the others is understood first. Consequently, studies on IA production from D-xylose have actually been performed decades ago (Eimhjellen and Larsen, 1955; Kautola et al., 1985; Kautola, 1990), and re-initiated in recent years (Saha et al., 2017, 2019; Wu et al., 2017; Saha and Kennedy, 2019).

Itaconic acid is formed by the same metabolic pathway as CA in *A. niger* – another bulk product of fungal primary metabolism – with the only exception that *A. terreus* performs thereafter two additional enzymatic steps (aconitate hydratase, *cis*-aconitate decarboxylase), leading to IA (Karaffa and Kubicek, 2019). The physiology and metabolic regulation of citric acid and IA formation are therefore very similar (Karaffa and Kubicek, 2003, 2019). The key parameters for obtaining high yields are a sucrose/glucose concentration of more than 100 g/L, a pH below 2.5, a high oxygen tension and a strict deficiency (<10^–7^ M) in Mn^2+^ ions. Some of them have been interpreted – at least in part – to an impact on the regulation of D-glucose catabolism and its energy balance (Karaffa and Kubicek, 2019). It is therefore not unreasonable to assume that these physiological requirements may also apply to IA production from D-xylose. There is a major difference, however: fungal catabolism of D-xylose occurs via the pentose catabolic pathway (Witteveen et al., 1989; Khosravi et al., 2018) and only at later stages feeds its intermediates into glycolysis. It is not known whether this alters the influence of the above parameters on IA production.

In this paper we have therefore tested whether two landmark nutritional requirements of the *A. terreus* IA overflow on D-glucose – Mn^2+^ ion deficiency and high concentration of the carbon source – also occur in a similar fashion on D-xylose as a sole source of carbon, and whether their respective optimization would give as molar yields close to the theoretical maximum from D-xylose as from D-glucose. We will show that the concentration of Mn^2+^ions and the carbon source indeed influence the concentration of IA produced, but that the maximally obtained molar yield of IA from D-xylose clearly stays below the theoretically possible one (0.63 vs. 0.83). We will therefore also provide carbon mass balances that show that a low biomass yield (*Y*_*x/s*_) from D-xylose is mainly responsible for the relatively lower molar IA yield even under the best fermentation conditions.

## Materials and Methods

### Fungal Strain and Cultivation Conditions

*Aspergillus terreus* NRRL 1960 (CBS 116.46; ATCC 10020), a standard high-producer strain was maintained on agar plates as described by Kuenz et al. (2012). Per litre of distilled water, the chemically defined minimal medium used throughout the experiments contained 0.1 g KH_2_PO_4_, 3 g NH_4_NO_3_, 1 g MgSO_4_ × 7 H_2_O, 5 g CaCl_2_ × 2 H_2_O, 1.67 mg FeCl_3_ × 6 H_2_O, 8 mg ZnSO_4_ × 7 H_2_O, and 15 mg CuSO4 × 7 H_2_O (Karaffa et al., 2015). The sole carbon source (D-xylose) and the Mn^2+^ ions were used at concentrations from 1 to 11% (w/v) and from <3 to 1000 μg L^–1^, respectively. To remove metal (particularly manganese) ion contaminations from the carbon source, D-xylose was dissolved in distilled water and passed through a column (440 × 45 mm) of Dowex 50 W-X8 (100/200) cation exchange resin. All components were added to this D-xylose solution from sterile stock solutions. The final Mn(II)-ion concentration was adjusted with MnCl_2_ × 4 H_2_O.

Shake-flask cultivations were performed in 500-mL Erlenmeyer flasks (VWR International Kft., Debrecen, Hungary) with 100 mL medium incubated at 33°C in rotary shaker (Infors AG, Basel, Switzerland) operating at 300 rpm, a rotation rate proven to provide sufficient aeration for IA overflow. The initial pH was set at 3.0 with 3 M HCl.

Bioreactor cultivations were carried out in 2.5-L glass fermentors (Sartorius AG, Göttingen, Germany) with a culture (working) volume of 2 L, equipped with one six-blade Rushton disk turbine impeller. Operating conditions were 33°C, and 0.75 vessel volume per minute (vvm) of aeration. Before inoculation, the pH was adjusted to 3.0 with 3 M HCl and not controlled during fermentation. Dissolved oxygen (DO) levels were maintained at 30% saturation by appropriately adjusting the impeller tip speed. DO, temperature, and impeller tip speed were controlled automatically by the regulatory units of the bioreactor. To minimize medium loss, the waste gas (from the headspace) was cooled in a reflux condenser connected to an external cooling bath (4°C) before exiting the system. Both shake-flask and bioreactor cultures were inoculated with 1 × 10^6^ *A. terreus* conidia per milliliter of medium from a freshly prepared, high-density spore suspension in a 1/10,000 Tween 20 solution.

All parts of the stirrer attachment as well as the aeration system and the sampling tube of the fermentors used in this study are built of stainless steel alloy that may contain up to 2% of manganese (Shackelford and William, 2001). The high temperature during sterilization, the acidic pH caused by organic acid accumulations and the sheer force inflicted by the mechanical agitation will inevitably corrode the steel surfaces and thus lead to the leaching of metal – including Mn(II) – ions into the growth medium. We therefore routinely checked the manganese ion concentrations after sterilization and during the fermentation process. When manganese ions were detected, fermentations were halted and the bioreactors were subjected to electrochemical polishing during which metal ions were thoroughly removed from the steel surfaces. Thus, leaching of manganese ions from the alloy of the fermentors did not interfere with the experiments.

All chemicals used were of analytical grade and purchased from Sigma-Aldrich (Budapest, Hungary).

### Analytical Methods

Volumetric mass transfer coefficients (*K_L_a*) of the shake-flasks at various rotation rates were estimated by the classic sulphite-oxidation method (Cooper et al., 1944), which relies on the conversion rate of a solution of sodium sulphite to sodium sulfate in the presence of 10^–3^ M copper(II) ions as catalysts.

Mycelial dry cell weight (DCW) was determined from 5 mL culture aliquots as described by Fekete et al. (2002). The biomass was harvested on a pre-weighted glass wool filter and washed with cold tap water, after which the filter was dried at 80°C until constant weight. Dry cell weight data reported in the section “Results” are the means of two separate measurements, which never deviated more than 14%.

Biomass yields (*Y*_*x/s*_) were calculated in two ways: the “overall yield” was calculated by relating the concentrations of the final biomass (DCW) to that of the total carbon source used (D-xylose). “Specific yield,” in contrast, was calculated by relating the concentrations of the final biomass to that of the consumed carbon source concentration from which the amounts needed for the biosynthesis of itaconic acid, CO_2_ and the identified side products were withdrawn. In the latter way, only the biochemical processes relevant to biomass synthesis and growth were considered.

The concentrations of D-xylose, itaconic acid, and α-ketoglutaric acid in the growth media were determined by high-pressure/performance liquid chromatography (HPLC; Gilson) with a proton exchange column (Bio-Rad Aminex HPX-87H^+^) at *T* = 55°C, using isocratic elution at 0.5 mL min^–1^ flow rate with 10 mM H_2_SO_4_ and refractive index (RI) detection. For xylitol determination, another ion exchanger column (Sarasep CAR-Ca), eluted with distilled water at 0.5 mL min^–1^ flow rate and *T* = 55°C was used, also with RI detection (Fekete et al., 2004). The concentrations were calculated from two independent measurements, which never deviated more than 5%. Maximal productivity (g IA L^–1^ h^–1^) was calculated from the biggest increase in IA concentrations between two subsequent samplings, while overall productivity was calculated with the period between time-point zero and the time-point at which IA concentration reached its maximum.

Manganese ion concentrations in the growth media were determined by inductively coupled plasma quadrupole mass spectrometry (ICP-QMS; Thermo Fisher Scientific, Bremen, Germany) equipped with Hexapole Collision Cell Technology (CCT), as described in details by Karaffa et al. (2015).

The determination of the overall fungal respiration rate was performed with an oxygraphic electrode (Strathkelvin Instruments Ltd., North Lanarkshire, United Kingdom) at 33°C, according to the manufacturer’s instructions. After oxygen consumption measurements, the biomass utilized in these assays was harvested and DCW was determined, allowing calculation of the specific oxygen uptake rates.

Fungal morphology was investigated by means of an Axio-Vision AC quantitative image analyser system. To increase contrast and visibility, lactophenol cotton blue (Fluka Chemie, Buch, Switzerland) was added to the samples in a final concentration of 10%. Stained samples were analyzed under a Zeiss AxioImager phase-contrast microscope, equipped with AxioCam MRc5 camera. Samples were taken at the early exponential phase (24 h) to study cell elongation. Later samples (48 h) were taken to assess the vacuolization and swelling of the cells.

### Reproducibility

All presented data are the means of three to five independent experiments. Data were analyzed and visualized with Sigmaplot software (Jandel Scientific), and for all cases standard deviations were determined. Quantitative data (*n* ≥ 3) were compared using ANOVA with Holm–Sidak Test for pairwise comparisons. While *p* values were often <0.001, the criterion for significance was *p* < 0.05 in all cases.

## Results

### Verification of the Experimental System

Because of the high number of independent fungal cultivations required for this analysis, we opted for shake-flasks as the cultivation method of choice in this study. However, to draw solid conclusions, we also performed controlled batch cultivations for those cultivations that appeared essential for the interpretation of the results in bench-scale fermentors (see Figure and Table legends).

To this end, to make sure that kinetics of fungal biomass, IA and residual D-xylose concentrations of the two cultivation systems would be independent of the vessel type (i.e., shake-flask or fermentor), we ensured that the physical parameters (temperature, pH, DO) were identical. Since the pH of an IA fermentation medium is adjusted at inoculation but not controlled further on, the most challenging task was to keep DO levels above 30% of saturation also in shake-flasks. To this end, we determined (a) the volumetric mass transfer coefficient (*K_L_a*) of the shake-flasks to be used for *A. terreus* cultivations, (b) the overall respiratory activity of the cultures (rX), and (c) the saturation concentration of oxygen (C_sat._) under the given experimental conditions in the growth media. From these data, actual oxygen concentrations (C_act._) could be calculated by the following equation (Bartholomew et al., 1950; Kozma and Karaffa, 1996):

$$C_{\mathrm{act}.}=C_{\mathrm{sat}.}-\mathrm{rX}/K_{L}\,a$$

Calculated actual oxygen concentrations in the shake-flasks both at the exponential and the stationary phases of the cultivation were found to be indeed high enough to provide sufficient aeration to IA overflow. As a consequence, maximal values of fungal biomass, IA or residual D-xylose concentrations as well as specific respiratory activity of the cultures grown in shake-flask or bioreactors – utilizing completely identical growth media – did not deviate by more than 10% at any of the time-points tested (Supplementary Table 1). We therefore considered our experimental system appropriate for the purposes of this study.

### Mass and Energy Balance of Itaconic Acid Formation From D-Xylose

**FIGURE 1:**
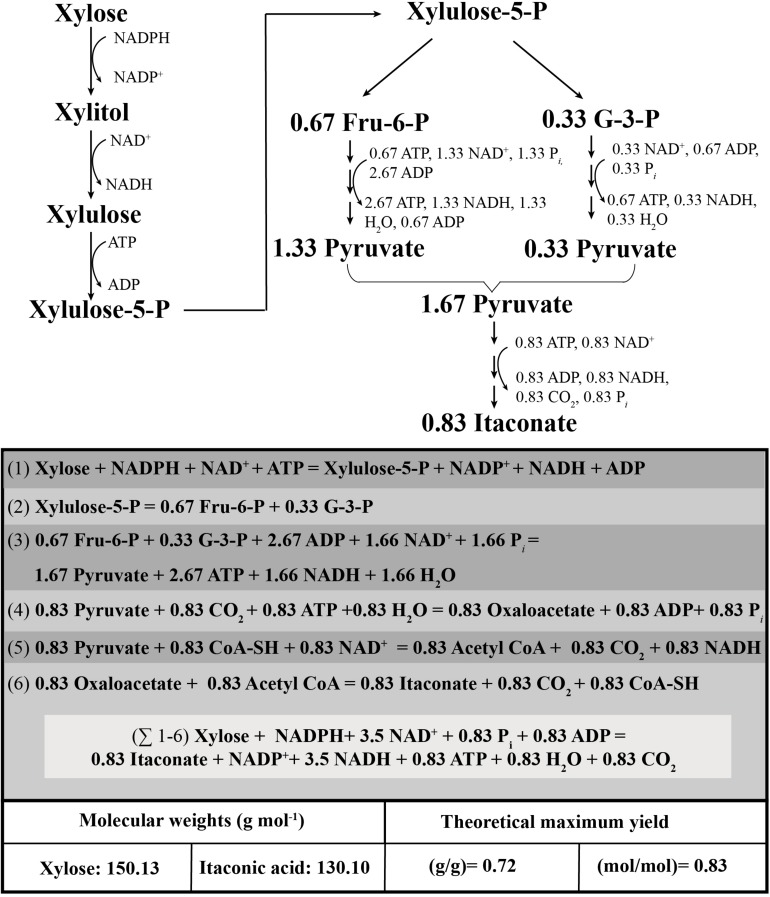
Co-factor and energy balance of fungal D-xylose catabolism leading to itaconic acid formation.

As a further prerequisite for this investigation, we first made a theoretical analysis of the possible yield of IA from the pentose D-xylose and the resulting energy gained and spent, based on the pentose catabolic known pathways in *A. terreus* (Aguilar-Pontes et al., 2018). Figure 1 shows the metabolic pathway from D-xylose to IA: xylose is first catabolized to xylulose-5-phosphate, which enters the pentose phosphate pathway. The correspondingly arising intermediates fructose-6-phosphate and glyceraldehyde-3-phosphate would then enter glycolysis. On a balance, 3 moles of D-xylose would thus be converted to 5 pyruvates with consequent gain of 8 NADH and 5 ATP. If we assume that the IA biosynthetic pathway would further commence as on D-glucose (i.e., by anaplerotic formation of oxaloacetate by pyruvate carboxylase), 2.5 moles of ATP would then be required for the pyruvate carboxylase reaction, thus reducing the ATP gain 2.5 moles per 3 moles of D-xylose. Shuttling of oxaloacetate into the mitochondria via conversion to malate and reconversion to oxaloacetate would be NADH-neutral but would exchange 2.5 moles of cytosolic NADH by 2.5 moles of mitochondrial NADH, which makes an important difference with respect to ATP generation by the respiratory chain (1.5 from cytosolic NADH, but 2.5 moles from mitochondrial NADH). In addition, the oxidative carboxylation of pyruvate to acetyl-CoA also generates 2.5 moles NADH per 3 moles of xylose. Therefore, the conversion of 1 mole of D-xylose to 1 mole of citrate (and thus IA) produces 0.83 moles of ATP, 1.83 moles of cytosolic NADH and 1.67 moles of mitochondrial NADH. The latter could be converted to further 6.92 moles of ATP, whose total amount is thus 7.75. This is somewhat lower than the 8.5 moles of ATP obtained from D-glucose, caused by a lower amount of ATP produced by substrate phosphorylation. The maximal amount of IA that can be obtained from D-xylose is therefore 0.83 (*Y*_*p/s*_; moles/moles) or 72 g per 100 g of D-xylose. Unless otherwise stated, we will use the molar *Y*_*p/s*_ throughout the manuscript.

### Itaconic Acid Production From D-Xylose Is Stimulated by Manganese Deficiency

**FIGURE 2:**
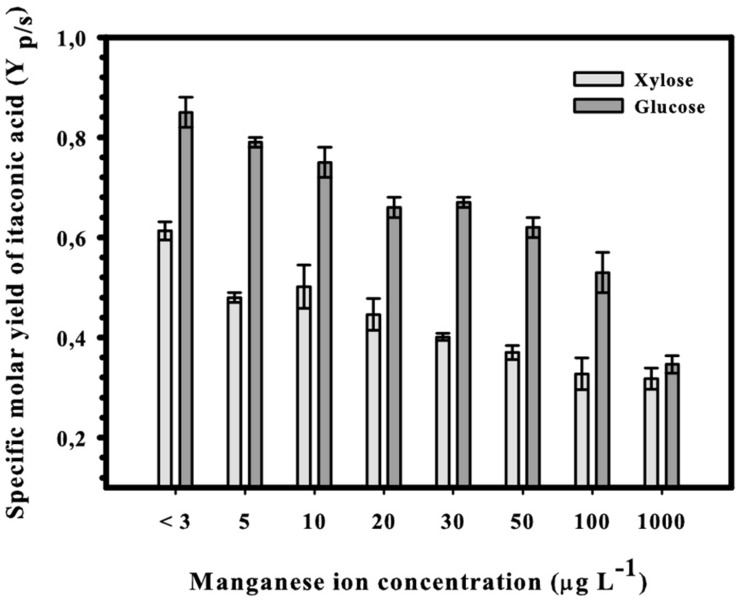
Final specific molar yields of itaconic acid in submerged shake-flask cultures of *Aspergillus terreus* NRRL 1960. The initial D-xylose concentration was 50 g L^– 1^ in itaconic acid-producing medium supplemented with different concentrations of Mn^2+^ ions.

We have reported recently that production of IA from glucose at concentrations close to the theoretical maximum requires a strict limitation of Mn^2+^ ions in the medium (Karaffa et al., 2015; Karaffa and Kubicek, 2019). We therefore tested whether this is also the case for IA production from D-xylose at a concentration of 50 g L^–1^. Figure 2 shows the result from this set of experiments: the highest molar yield (*Y*_*p/s*_ = 0.63) of IA was indeed obtained at cultivation below 3 μg L^–1^ Mn^2+^, which represents 75% of the theoretical maximum molar yield given above. Increasing the concentration of Mn^2+^ ions up to 100 μg L^–1^ results in an approximately 50% reduction of the IA yield, which is comparable to what has been published for D-glucose (Karaffa et al., 2015). High production of IA from D-xylose therefore also requires a deficiency of Mn^2+^ in the medium.

### The Morphology of D-Xylose Grown *A. terreus* Cultures Is Dependent on the Concentration of Mn^2+^

**FIGURE 3:**
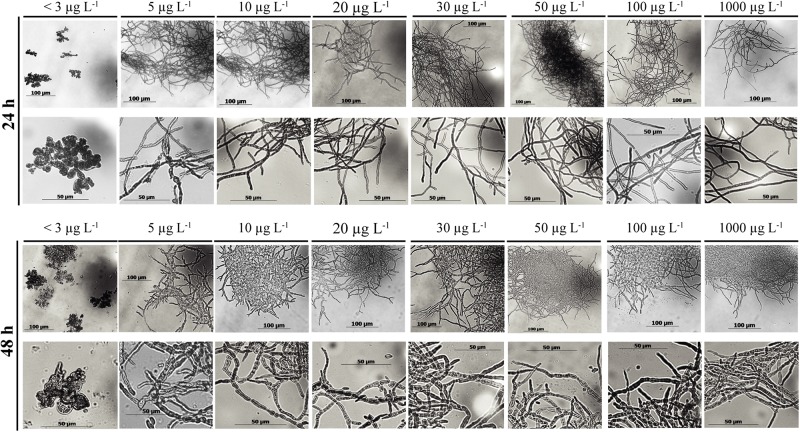
Microscopic images of submerged shake-flask cultures of *Aspergillus terreus* NRRL 1960. The initial D-xylose concentration was 50 g L^– 1^ in itaconic acid-producing medium supplemented with different concentrations of Mn^2+^ ions. Images taken at 24 and 48 h, respectively.

**FIGURE 4:**
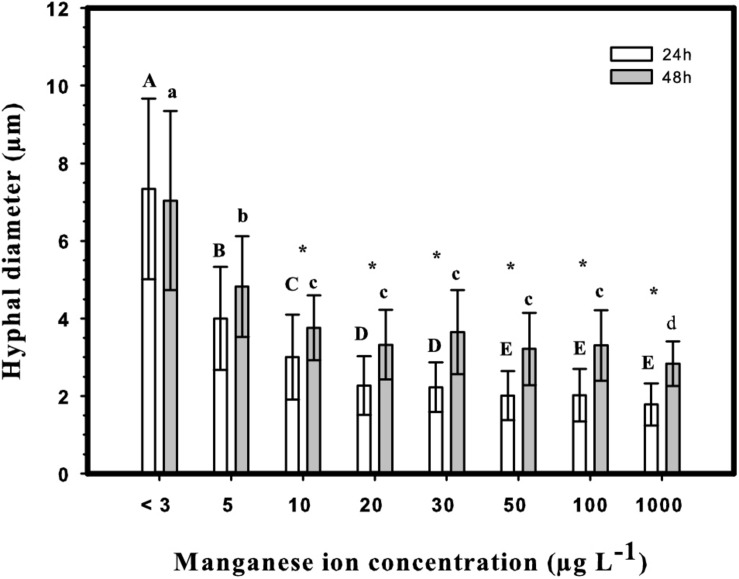
Hyphal diameters at 24 h (white columns) and 48 h (gray columns) after inoculation in shake-flask cultures. Upper case letters: significantly different groups at 24 h (*p* < 0.05). Lower case letters: significantly different groups at 48 h (*p* < 0.05). Asterisks indicate significant changes between 24 and 48 h in the same culture (*p* < 0.05). Statistical analysis was performed using ANOVA with Holm–Sidak Test for pairwise comparisons.

The morphology of a fungal culture has an important impact on its behavior in submerged cultivation because it influences the surface available for nutrient transport and the viscosity of the medium. A deficiency in manganese ions is known to change the morphology of *Aspergillus* spp. from smoothly filamentous to stubbed, swollen, highly branched and even yeast-like hyphae (Barnett and Lilly, 1966; Garrison and Boyd, 1974; Kisser et al., 1980; Papagianni, 2006; Clark et al., 2016). In agreement with this, the morphology of *A. terreus* at Mn^2+^ concentration <3 μg L^–1^ was mostly represented by yeast-like forms and loose cell clumps, and formation of hyphae occurred only over 5 μg L^–1^ (Figure 3). Although hyphal morphology was dominant already at 5 μg L^–1^, some yeast-like cells could still be detected, but disappeared completely at Mn^2+^ concentrations > 5 μg L^–1^. In line with this, the cell diameters decreased gradually and significantly with increasing Mn^2+^ concentrations in the growth medium (Figure 4), the same behavior also observed with the IA yield (Figure 2).

Interestingly, cell diameters significantly increased between the 24 and 48 h of cultivation in every culture with Mn^2+^ concentration >10 μg L^–1^, while they remained constant in cultures with the two lowest (<3 and 5 μg L^–1^) concentrations. No further swelling of cells was observed in cultures older than 48 h. Summarizing, similarly to the case on D-glucose, morphology of D-xylose grown *A. terreus* cultures – quantified as cell diameter – is extremely sensitive to the changes in the Mn^2+^ ion concentration in the medium, even within a range considered entirely suboptimal for growth (from <3 to 10 μg L^–1^).

### The Highest Molar Yield of Itaconic Acid From D-Xylose Is Already Reached at 50 g/L

**FIGURE 5:**
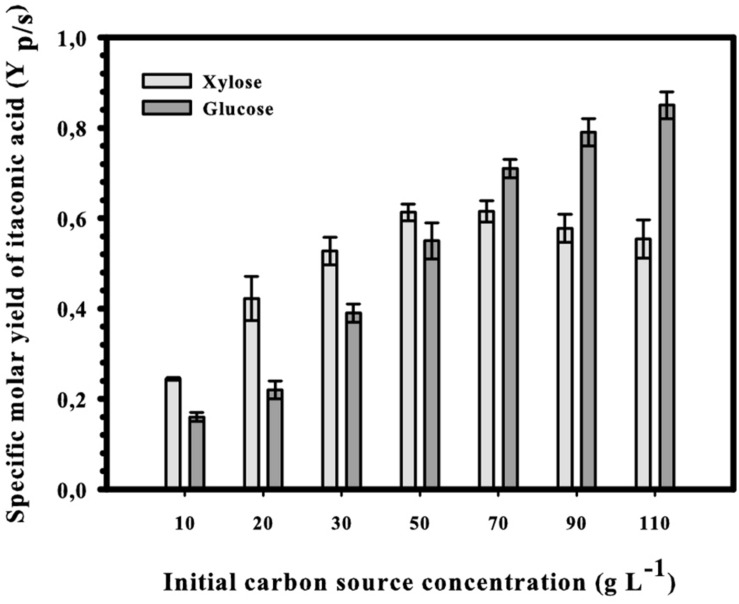
Specific molar yield of itaconic acid at different initial D-xylose concentrations in Mn^2+^ ion-limited shake-flask cultures of *Aspergillus terreus* NRRL 1960. Yields were calculated from the consumed D-xylose and maximal achieved IA concentrations.

**FIGURE 6:**
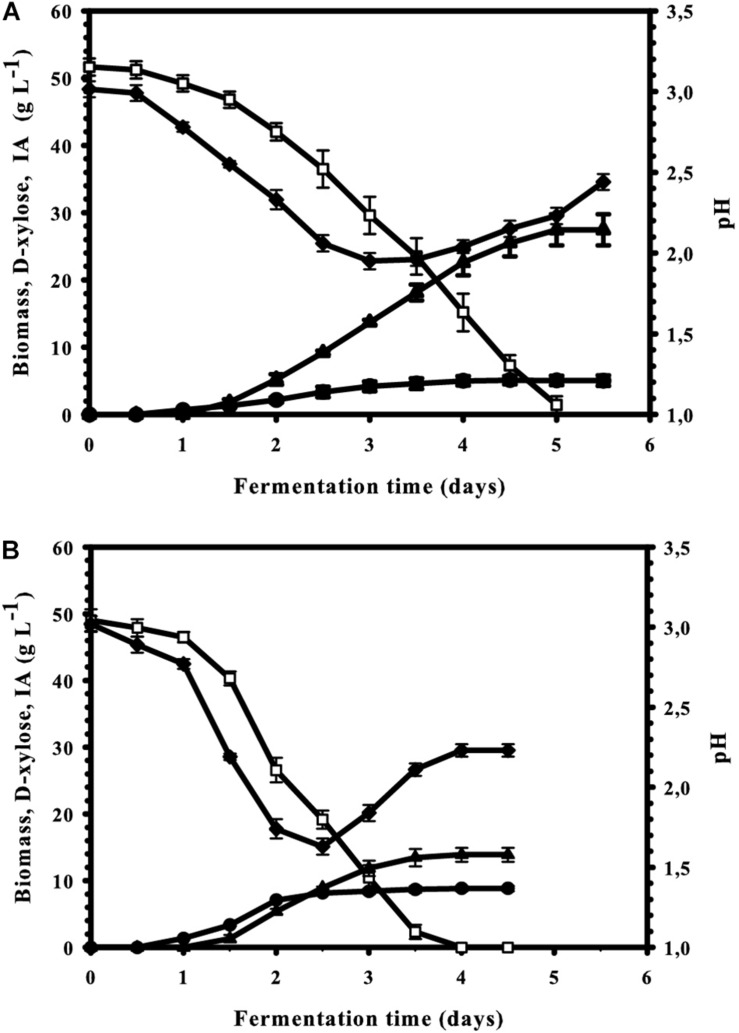
Kinetics of biomass (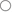), itaconic acid (▲), and residual D-xylose (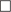) concentrations as well as of the medium pH (◆) in controlled batch fermentations of *A. terreus*, performed in a 2-L scale bioreactor. The first three variables share the same *y*-axis as they are expressed in the same unit (g L^– 1^). **(A)** Mn^2+^ ion-limited culture (<3 μg L^– 1^]. **(B)** Culture supplemented with 0.1 mg L^– 1^ Mn^2+^ ions.

The second critical parameter for IA production from D-glucose is the concentration of the carbon source, and maximal yields are obtained only at concentrations over 120 g L^–1^ (Karaffa et al., 2015). We therefore tested whether the optimal concentration of D-xylose would also be in this range. The results shown in Figure 5: there is indeed a steady increase in IA formation when the D-xylose concentration is raised, resulting a plateau already at in a maximal concentration of 54 g L^–1^ IA at 11% (w/v) xylose. This corresponds to a molar yield of 55% and represents 73% of the theoretical maximum (83%; see Figure 1). The maximal and overall volumetric productivity under these conditions were 0.37 and 0.23 g IA L^–1^ h^–1^, respectively. Interestingly, and in contrast to the findings with D-glucose, this highest molar yield (63%) is already reached at 5% (w/v) D-xylose. The *Y*_*p/s*_ at 11% (w/v) is lower than at 5% (w/v), although the difference is statistically not significant (*p* = 0.32). However, irrespective of the initial concentration of D-xylose, kinetics of four fundamental fermentation parameters – pH, and concentration of biomass, D-xylose and IA were qualitatively similar to the one displayed at Figure 6. [i.e., 5% (w/v) initial D-xylose and Mn^2+^ concentration <3 μg/L]. Consumption of D-xylose paralleled IA formation.

### Biomass and By-Product Formation During IA Production on D-Xylose

The conversion of only about 50% of the available and fully consumed D-xylose into IA raises the question about the fate of the remaining carbon. One point to be considered here is the release of 1 mole of carbon dioxide per 1 mole of IA by the *cis*-aconitate decarboxylase (CAD) reaction. A calculation shows that this accounts for up to 18.2 g L^–1^ on 11% (w/v) xylose (Table 1). We also checked for the presence of xylitol, which is a common by-product of fungal growth in D-xylose (Bruinenberg et al., 1984; Weyda et al., 2014), but found only very low concentrations (∼0.4 g L^–1^; corresponding to ∼8 mmoles/mole D-xylose). We further detected small amounts of α-ketoglutarate in the medium and they increased from 0.3 to 1.2 g L^–1^ when the concentration of D-xylose was raised from 5 to 11% (w/v), which resembles 11 mmoles/mole D-xylose (Table 1). No other extracellular carbon components were detected. Consequently, we can rule out the formation of by-products as a reason for the gap between the theoretical and the achieved maximal molar yield of IA.

**TABLE 1:** Biomass (DCW) and itaconic acid (IA) production as well as derived kinetic parameters of *Aspergillus terreus* NRRL 1960 cultivations as a function of the initial D-xylose concentration.

**Initial D-xylose (g L^–1^)**	**Maximal IA (g L^–1^)**	**Final DCW (g L^–1^)**	**Xylitol (g L^–1^)**	**α-ketoglutarate (g L^–1^)**	**CO_2_ from CAD reaction (g L^–1^)**	**IA molar yield (*Y*_*p/s*_)**	**Overall cell mass yield^1^ (*Y*_*x/s*_)**	**Specific cell mass yield^2^ (*Y*_*x/s*_)**
**9.79 ± 0.71**	2.08 ± 0.17	3.17 ± 0.1	–	–	0.7	0.24	0.32	0.45
20.15 ± 2.49	7.33 ± 0.85	3.85 ± 0.3	0.01 ± 0.01	0.02 ± 0.02	2.48	0.42	0.19	0.37
31.81 ± 1.99	14.57 ± 1.77	4.67 ± 0.4	0.02 ± 0.01	0.12 ± 0.08	4.93	0.53	0.14	0.38
								

**51.65 ± 1.26**	27.45 ± 1.32	4.93 ± 0.4	0.05 ± 0.01	0.32 ± 0.14	9.28	0.63	0.1	0.34

69.97 ± 0.26	37.29 ± 1.32	7.46 ± 0.9	0.10 ± 0.01	0.49 ± 0.12	12.62	0.62	0.1	0.37
91.28 ± 1.04	45.71 ± 2.85	8.01 ± 0.2	0.21 ± 0.02	0.73 ± 0.10	15.46	0.58	0.09	0.27
**110.46 ± 0.74**	53.97 ± 2.68	8.49 ± 0.6	0.37 ± 0.02	1.15 ± 0.14	18.25	0.55	0.08	0.23

*Mycelia grew under submerged conditions in shake-flasks in an optimized IA-producing medium (see section “Materials and Methods”). Cultivations with 10, 50, and 110 g/L initial D-xylose concentrations (indicated by bold numbers) were reproduced in 2-L scale bioreactors under controlled batch conditions (see section “Results”). Framed dataset served as a basis for Figure 6. ^1^Calculated by relating the concentrations of the final biomass (DCW) to that of the total supplied carbon source (D-xylose). ^2^Calculated by relating the concentrations of the final biomass (DCW) to that of the total supplied carbon source (D-xylose), from which the amounts needed for the biosynthesis of the side products, CO_2_ and itaconic acid were deduced (i.e., only processes relevant to biomass synthesis and energy required for growth were considered).*

Because of these findings, the remaining gap in the carbon used and products found must be accounted for by biomass formation. We found that under optimized IA producing conditions – that are obviously suboptimal for growth – the biomass yield (g per g carbon source present) for D-xylose was 0.1 at 5% (w/v) and 0.08 at 11% (w/v) initial concentrations (Table 1). In contrast, the biomass yield was reported to be 0.14 (w/w) for D-glucose (Molnár et al., 2018) under otherwise identical conditions. However, when the biomass yield is calculated only on the basis of the remaining carbon (i.e., after eliminating the carbon needed for IA and side-product formation), cultivation of *A. terreus* at 1% (w/v) D-xylose led to a yield of *Y*_*x/s*_ = 0.45 (Table 1). This specific biomass yield gradually declines with increasing D-xylose concentration: at 11% (w/v), the IA concentration formed is 54 g L^–1^, the carbon dioxide removed by CAD is 18.2 g L^–1^, and thus the *Y*_*x/s*_ is only 0.23 (Table 1). We therefore conclude that IA is indeed the only product of cultivation on D-xylose, and its lower molar yield is due to a high carbon demand for biomass production.

## Discussion

In this paper, we have tested the hypothesis that the application of two key parameters for obtaining IA by submerged fermentation from D-glucose in high yields – a deficiency in Mn^2+^ ions and a carbon source concentration of >100 g/L – would also result in yields of IA from D-xylose close to the theoretical maximum. Indeed, our results confirmed the need for Mn^2+^ ion deficiency, but rejected the hypothesis that the carbon source concentrations must be as high as on D-glucose. Notably, the maximal concentration of IA formed under the highest yielding conditions was only two third of the theoretical maximum.

The above findings support the interpretation that the effect of Mn^2+^ ions is independent of the carbon source and therefore rather due to an interaction either with transport and/or improved rheology and aeration via the compact pellet morphology (Rychtera and Wase, 1981; Okabe et al., 1993), whereas the need for a high D-glucose concentration is specific to D-glucose metabolism. In *A. niger*, the increase of D-glucose over 10% has been reported to induce pyruvate carboxylase and the formation of fructose-2,6-bisphosphate, a key regulator of glycolysis at the level of phosphofructokinase (PfkA), and which can relieve the inhibition of the enzyme by high citrate concentrations (Kubicek et al., 1990). We do not know whether an induction of pyruvate carboxylase of *A. terreus* by higher D-xylose concentrations occurs, but the flux through PfkA and the later stages of glycolysis theoretically accounts for only two of three D-xylose molecules (cf. Figure 1) and therefore is less important for the flux to pyruvate. It is important to note that an increase in the xylose concentration up to 11% (w/v) also increases the final concentration of IA (because of the provision of more substrate) but – unlike the situation on glucose – does not increase the yield *Y*_*p/s*_. In fact, there is even a small decrease in *Y*_*p/s*_ at concentrations over 7% (w/v). Yet this loss of carbon at 11% (w/v) is not significantly different from that at 5% (w/v) D-xylose, and working at high D-xylose concentration (maybe even higher than the ones used in this work) can be used to produce high concentrations of IA without major negative effects on the economy of the process.

Despite of optimizing these two conditions, the yield of itaconic acid from D-xylose lies significantly below the theoretically possible yield. The formation of other products besides IA (such as xylitol) would have been the most likely cause for this. However, we could show that *A. terreus* does not form byproducts in significant amounts; therefore, the poor energy balance of growth on D-xylose is the most plausible explanation (Figure 1). Metabolic control analysis has suggested that the NADPH-dependent aldose reductase exerts main flux control during the D-xylose catabolic pathway in Aspergilli (Prathumpai et al., 2003). Our findings that no significant accumulation of xylitol takes place during growth of *A. terreus* on D-xylose suggests that the enzyme may indeed limit the rate of flux, what could be due to a limiting concentration of NADPH. One enzyme that could enhance the NADPH/NADH ratio would be NADH-kinase, whose presence has been demonstrated in *A. nidulans* (Panagiotou et al., 2009), and whose overexpression enhanced growth on D-glucose. Yet we consider NADH-kinase as a less likely candidate because it would consume more than half of the NADH generated in the cytosol and thus further reduce the energy gained from D-xylose. Other enzymes forming NADPH are the oxidative enzymes of the pentose phosphate pathway (glucose-6-phosphate dehydrogenase and 6-phosphogluconate dehydrogenase) as well as the NADPH-dependent isocitrate dehydrogenase. The latter has been proposed to play the more significant role in *A. nidulans* (Singh et al., 1988), and our findings of albeit small amounts of α-ketoglutarate in the medium suggests that this may also be valid for *A. terreus*. NADPH is predominantly used for anabolic purposes and its withdrawal by aldose reductase thus reduces the formation of metabolites necessary for cell growth. In addition, the above enzymes will also affect the yield of itaconic acid by withdrawing carbon dioxide or *cis*-aconitate, respectively, from itaconate biosynthesis. Our data and these considerations therefore strongly suggest that an increase in *Y*_*x/s*_, the yield coefficient of biomass formation from D-xylose, would be a tool for further increasing the yield of itaconic acid from D-xylose.

Which strategies could be used to improve *Y*_*x/s*_? As explained above, manipulation of the NADPH pool would likely be counterproductive. Panagiotou et al. (2008) reported that overexpression of a phosphoketolase encoded by the *phk* gene increased the *Y*_*x/s*_ on D-xylose from 0.19 to 0.46. This can be explained by the energy balance of catabolism of D-xylose via the phosphoketolase pathway: as we have shown above (Figure 1), catabolism of D-xylose via the canonical pathways yields 0.83 moles of ATP per mole of xylulose-5-phosphate. Phosphoketolase, however, cleaves xylulose-5-phosphate directly to glyceraldehyde-3-phosphate and acetyl-phosphate, which results in the formation of 2 ATP from the triose phosphate catabolism to pyruvate. Yet per 1 mole of xylose, 1 ATP is consumed by the formation of xylulose-5-phosphate, and one by the pyruvate decarboxylase reaction. So the ATP balance would be zero. The balance of NADH is more difficult, however: 1 NADH is gained by the conversion of xylitol to L-xylulose, and a further one by the glyceraldehyde-3-phosphate dehydrogenase reaction. The cytosolic generation of acetyl-CoA from acetyl-phosphate, however, would bypass the generation of 1 mole NADH/D-xylose in the pyruvate dehydrogenase reaction. Also, the malate shuttle of oxaloacetate, which would increase the ATP yield from 1 NADH will likely not occur: the operation of a cytosolic pathway of IA biosynthesis has recently been proposed by the finding of a cytosolic citrate synthase CitB in *A. terreus*, whose overexpression stimulates IA formation (Hossain et al., 2016; Karaffa and Kubicek, 2019). Therefore, a combination of the phosphoketolase pathway with cytosolic citrate (and thus IA) biosynthesis would generate no ATP and 2 NADH from xylose. This pathway therefore generates significantly less energy than the canonical pathway via the lower part of glycolysis.

Why this pathway therefore enhances *Y*_*x/s*_ during D-xylose catabolism (Panagiotou et al., 2008) cannot therefore be explained on the basis of gained energy. Alternatively, the phosphoketolase pathway would omit the operation of the pentose phosphate pathway for D-xylose catabolism and thus save its intermediates for anabolism. In fact, transformation of *A. nidulans* with its phosphoketolase gene reduced the operation of the pentose phosphate pathway by 22% (Panagiotou et al., 2008).

Summarizing, our study shows that the application of Mn^2+^ ion deficiency (like in citric acid fermentation) leads to the accumulation of IA as the sole fermentation product from D-xylose, and that the use of high pentose concentrations increases IA formation, but neither stimulates nor affects its yield *Y*_*p/x*_. We also show that the biomass yield *Y*_*x/s*_ is likely limiting an increase in *Y*_*p/s*,_ and its manipulation would therefore be the preferred strategy to improve IA accumulation. Our findings can probably also be applied to IA production from L-arabinose, the other pentose occurring in lignocellulose.

## Data Availability

All datasets generated for this study are included in the manuscript and/or the Supplementary Files.

## Author Contributions

IK and LK conceived the study. IK, ÁM, and LK designed the experiments. IK, ÁM, and ES performed the experiments. All authors analyzed the data, read, and approved the final manuscript. BK contributed to the essential analysis tools. LK supervised the experimental work and data analysis. LK and EF wrote the grant proposals and obtained funding. IK, ÁM, EF, CK, and LK wrote the manuscript.

## Conflict of Interest Statement

The authors declare that the research was conducted in the absence of any commercial or financial relationships that could be construed as a potential conflict of interest.
