# Supplementary material for: Manganese Deficiency Is Required for High Itaconic Acid Production From D-Xylose in Aspergillus terreus
Source: Front Microbiol. 2019 Jul 9;10:1589. doi: 10.3389/fmicb.2019.01589 (PMC6629873; doi:10.3389/fmicb.2019.01589)
Supplement: Supplementary file 1 [file Data_Sheet_1.pdf]

Supplementary Material

Manganese deficiency is required for high itaconic acid production from D-xylose in *Aspergillus terreus*

István S. Kolláth[1], Ákos P. Molnár[1], Áron Soós[2], Erzsébet Fekete[1], Erzsébet Sándor[2], Béla Kovács[2], Christian P. Kubicek[3] and Levente Karaffa[1*]

[1]Department of Biochemical Engineering, Faculty of Science and Technology, University of Debrecen, Debrecen, Hungary

[2]Institute of Food Science, Faculty of Agricultural and Food Science and Environmental Management, University of Debrecen, Debrecen, Hungary

[3]Institute of Chemical, Environmental & Bioscience Engineering, TU Wien, Vienna, Austria

*** Correspondence:**
Dr. Levente Karaffa
levente.karaffa@science.unideb.hu

Supplementary Table 1. Specific respiration rates (µM min^{-1} $g_{DCW}$$^{-1}$) of *Aspergillus terreus* NRRL 1960 cultures grown in 500-mL shake-flasks and 2-L bioreactors (fermentors) in IA-producing minimal medium with 1, 5 and 11% (w/v) initial D-xylose as the sole carbon source. Samples for assays were taken at the rapid growth and the stationary phase. Mn^{2+} concentrations were below 3 µg/L^{-1} in all cases.

Initial D-xylose (g L^{-1})	Shake-flask cultures		Bioreactor cultures	
	rapid growth phase	stationary phase	rapid growth phase	stationary phase
9.9 ± 0.8	28.6 ± 1.0	10.2 ± 0.8	29.1 ± 1.1	9.6 ± 1.1
51.1 ± 1.4	28.1 ± 1.3	9.3 ± 0.7	27.4 ± 1.2	10.2 ± 1.0
110.1 ± 0.9	29.6 ± 1.5	9.9 ± 1.0	30.4 ± 1.6	10.6 ± 1.3

.
